# The evolving therapeutic landscape of genetic skeletal disorders

**DOI:** 10.1186/s13023-019-1222-2

**Published:** 2019-12-30

**Authors:** Ataf Hussain Sabir, Trevor Cole

**Affiliations:** West Midlands Clinical Genetics Unit, Birmingham Women’s and Children’s NHS FT and Birmingham Health Partners, Birmingham, UK

**Keywords:** Skeletal, Bone, Dysplasia, Rare, Genetic, Hereditary, Therapy, Personalised, Targeted

## Abstract

**Background:**

Rare bone diseases account for 5% of all birth defects yet very few have personalised treatments. Developments in genetic diagnosis, molecular techniques and treatment technologies however, are leading to unparalleled therapeutic advance. This review explores the evolving therapeutic landscape of genetic skeletal disorders (GSDs); the key conditions and there key differentials.

**Methods:**

A retrospective literature based review was conducted in December 2018 using a systematic search strategy for relevant articles and trials in Pubmed and clinicaltrials.gov respectively. Over 140 articles and 80 trials were generated for review.

**Results:**

Over 20 personalised therapies are discussed in addition to several novel disease modifying treatments in over 25 GSDs. Treatments discussed are at different stages from preclinical studies to clinical trials and approved drugs, including; Burosumab for X-linked hypophosphatemia, Palovarotene for Hereditary Multiple Exostoses, Carbamazepine for Metaphyseal Chondrodysplasia (Schmid type), Lithium carbonate and anti-sclerostin therapy for Osteoporosis Pseudoglioma syndrome and novel therapies for Osteopetrosis. We also discuss therapeutic advances in Achondroplasia, Osteogenesis Imperfecta (OI), Hypophosphotasia (HPP), Fibrodysplasia Ossificans Progressiva, and RNA silencing therapies in preclinical studies for OI and HPP.

**Discussion:**

It is an exciting time for GSD therapies despite the challenges of drug development in rare diseases. In discussing emerging therapies, we explore novel approaches to drug development from drug repurposing to in-utero stem cell transplants. We highlight the improved understanding of bone pathophysiology, genetic pathways and challenges of developing gene therapies for GSDs.

## Introduction

Rare bone diseases account for 5% of all birth defects and are an important cause of disability Worldwide, yet they remain a difficult group of conditions to treat [1]. In the 2015 *Nosology and Classification of Genetic Skeletal Disorders (GSDs)* there were 436 GSDs with hundreds of causative genes [2]. Improved understanding of the fundamental pathophysiological pathways in GSDs has accelerated interest in related personalised therapies.

Previous reviews of therapeutic advances in skeletal disorders; such as Yap et al. in 2016, Jelin et al. and Nikkel in 2017, have focused on the well-known rare conditions; Achondroplasia (ACH), Osteogenesis Imperfecta, Hypophosphatasia, Fibrodysplasia Ossficans Progressiva (FOP) and Mucopolysaccharidoses, (MPS, types; I-II, IVa, VI) [3–5].

Since then, further therapies have developed for other skeletal disorders, such as; Hereditary Multiple Exostoses (HME), Metaphyseal Chondrodysplasia Schmid type (MCDS), Osteopetrosis, X-linked hypophosphatemia (XLH), MPS IIIB (Sanfillippo B) and MPS VII (Sly), as well as expanding treatments in ACH and OI [6].

For clinicians, to keep abreast of such advances is difficult. This article reviews the evolving therapeutic landscape of GSDs, discussing those GSD’s with targeted therapies in development or use, explores the genomic basis of the conditions / therapy and where relevant, discusses clinical diagnoses, considering the main differentials. It is important to ‘know’ the conditions and their subtleties so that variants from future gene panels, exome /genomic sequencing can be evaluated accurately, as well as being able to recognise new phenotypes.

## Methods

This article is a retrospective literature based review. Using Pubmed we searched for English language papers during December 2018 using the following search terms: ‘skeletal dysplasia / dysplasia’ or ‘genetic skeletal / bone’ or ‘inherited skeletal or bone’ AND ‘treatment’ or ‘therapeutic’ or ‘personalised therapy / treatment’ or ‘targeted therapy / treatment’. Over 140 abstracts were reviewed, and not restricted by publication type (e.g. case report). All abstracts greater than 15 years old were excluded as beyond this period very few relevant treatments existed. Remaining abstracts were screened for clinical relevance. The emphasis of this review was to consider personalised therapies and drugs that modify the underlying disease process, thus abstracts discussing orthopaedic / surgical interventions were excluded. Non-genetic conditions were also excluded.

A second strategy to uncover therapies that may have not yet been published or treatments that may have been missed from the Pubmed search, was to search the clinicaltrials.gov website using the same search strategy with an additional filter for ‘intervention’ type studies. Over 80 trials were identified in December 2018 and reviewed for clinical relevance.

**Fig. 1:**
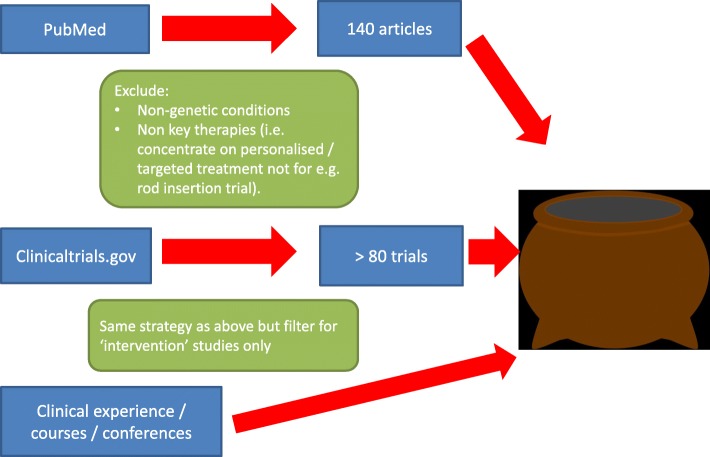
Flow diagram illustrating search strategy

Lastly any new therapies learnt about through conferences, study days, word of mouth etc. have also been included (Fig. 1).

## Results

### FGFR3 spectrum disorders

Fibroblast growth factor receptor 3 gene (*FGFR3*) mutations lead to group of autosomal dominant disorders that in descending severity include; Thanatotrophic dysplasia (TD), Achondroplasia (ACH) and Hypochondroplasia (HCH). FGFR3 negatively regulates endochondral ossification, thus activating *FGFR3* mutations limit endochondral growth, leading to disproportionate short stature. Permanent activation of FGFR3 signalling activates two intracellular signalling cascades; STAT-1 (signal transducer and activator of transcription, leading to decrease chondrocyte conversion to bone) and MAPK (mitogen-activated protein kinase, leading to decreased production of extracellular matrix (ECM)) [7].

TD is one of the commonest lethal skeletal disorders. Patients present with severe micromelia, macrocephaly with frontal bossing and reduced thoracic volume [8].

ACH is the commonest post natal cause of disproportionate short stature. Unlike with TD, in ACH limb shortening is less severe and is rhizomelic, with redundant skin folds, reduced hip and elbow rotation, and metaphyseal flaring. Characteristic facial features include; macrocephaly, frontal bossing, a depressed nasal bridge, crowded teeth, mid-facial hypoplasia giving the impression of jaw protrusion, along with trident shaped hands, genua vara and brachydactyly [9]. ACH is associated with a number of orthopaedic, neurological, respiratory and ENT complications such as foramen magnum stenosis, craniocervical instability, sleep apnoea, restrictive and obstructive lung disease, scoliosis, spinal stenosis, recurrent ear infections, chronic pain and obesity. It has an incidence of 1 in 25,000 live births and is denovo in 80% of cases. Approximately 97% of ACH cases are caused by a G380R substitution of *FGFR3*, resulting in ligand dependent gain of function (through FGFR3 dimer stabilisation), though there are differing positions as to the biological mechanism which we discuss below [10].

The presentation of HCH is milder than ACH (with less pronounced limb disproportion and short stature and milder radiological findings). Life expectancy for individuals with HCH is normal, whereas historical literature suggests lifespan in ACH is reduced by 10 years on average due to the fore-mentioned complications (particularly in children under 4 years old due to brainstem compression leading to sudden death) [11, 12]. Recent literature suggests lifespan to be nearer to normal due to better surveillance and newer treatments [13]. Around 70% of HCH cases are caused by FGFR3 mutations (with the cause unknown in the remainder, though the question remains whether these cases really are HCH) and over 90% of these are due to the N540K mutation (C > A in ~ 70%, C > G in ~ 30%). Over 20 other mutations across FGFR3 are known including; N540 T, N540S and I538V [14]. Growth hormone (GH) is used in HCH (Table 1).

**Table 1:** *FGFR3* gene related skeletal disorders and relative differences

FGFR3 Spectrum disorders		
Thanatotrophic Dysplasia	Achondroplasia	Hypochondroplasia
Perinatally Lethal Severely disproportionate limb short stature, narrow chest with short ribs, underdeveloped lungs, macrocephaly,	Reduced lifespan* (see above text) Disproportionate limb short stature, brachydactyly, macrocephaly with prominent forehead, mid-facial hypoplasia,flattened nasal bridge, kyphosis / lordosis, varus / valgus deformities	Normal Lifespan Mild disproportionate limb short stature, increased head circumference, normal facies.
Commonest mutation(s); R248C and K650E [13]	G380R	N540K

### Pharmacological agents for Achondroplasia: Vosoritide (CNP analogue) and TransCon CNP

Though there are currently no approved FDA treatments for ACH, Biomarin has developed Vosoritide (BMN111), a C-type natriuretic peptide (CNP) analogue derived from natural human peptide. Naturally occurring CNP has a short half-life due to enzyme degradation whereas BMN-111 resists enzyme proteolytic activity. CNP is a known negative regulator of FGFR3. It binds natriuretic-peptide receptor 2 (NPR2) inducing cyclic gaunosine-3′.5′ monophosphate (cGMP) synthesis from Guanosine-5′-triphosphate (GTP), thus inhibits the MAPK pathway [15, 16].

Vosoritide acts to increase ECM production, which along with chondrocytes act as a template for enchondral ossification. The STAT1 pathway has not been affected though.

Phase 2 results for Vosoritide demonstrated favourable safety profiles and an increase in annual growth velocity of 50% (following daily subcutaneous injections at 15 μg/kg for 12 months) compared to pre-treatment growth velocity [17]. Further results from the phase 2 study published in July 2019 showed a generally mild side effect profile in the 35 study participants, 30 completed the study; 2 patients withdrew due to fear of needles. Sustained annualised growth velocity was demonstrated for up to 42 months at the 15 μg/kg and 30 μg/kg doses [18]. Phase 3 clinical trials are currently under way in children aged 5–18 years at the 15 μg/kg dose with UK recruitment centres in Sheffield and London ((NCT03197766). A phase 2, trial is ongoing (NCT03583697) of Vosoritide in infants and younger children (age range, 0 to < 60 months).

Though Vosoritide is a major breakthrough as compared to the standard of care, there is concern with respect to long term results. Some patients with ACH have elevated levels of CNP suggesting natural resistance may be an issue. Additionally subcutaneous daily injections are not ideal [19]. The short life of Vosoritide leads to high peak-to-trough ratios with hypotension seen in some patients coinciding with maximal blood concentrations indicating a causal relationship [20].

The body proportions (upper body length to leg length ratio) showed no improvement with Vosoritide treatment in the phase 2 trial, perhaps because the STAT1 pathway is not manipulated.

Ascendis Pharma have developed a slow release CNP analogue (TransCon CNP) where the CNP is bound to and shielded by a TransCon Carrier. Carrier autohydrolysis allows for sustainable release over 7 days. A phase 1 trial in healthy adults has been completed and a phase 2 trial is planned in 2019 Q3.

### Pharmacological agents for Achondroplasia: TA-46

One drug in particular that aims to improve anatomical proportions is TA-46. TA-46 is a recombinant FGFR3 ligand trap (a soluble form of human FGFR3), initially developed by Therachon AG [21].

Early literature has long held that ACH is caused by ligand-independent FGFR3 activation, so why would a ligand trap be useful? [22] More recent work has suggested that dimerization and over activation of FGFR3 are ligand-dependant and the discrepancy between the earlier and later work may be due to differing cellular systems, though there is no clear consensus in the literature suggesting multiple mechanisms leading to prolonged intracellular signalling [23].

TA-46 binds to FGFR3’s natural ligands, preventing the ligands from binding to the FGFR3 receptor / over-activating the STAT1 and MAPK pathways. Thus TA-46 is effectively a decoy receptor or a ‘ligand trap’. TA-46 thus normalises overactive FGFR3 signalling at a more root level. TA-46 has received orphan drug designation by the EMA (European Medical Agency) and FDA (US Food and Drug Administration agency) in 2018 and phase 1 studies have been completed with phase 2 studies planned for late 2019 [24]. Studies of sFGFR3 (soluble FGFR3 protein) in ACH mouse models have demonstrated symptom rescue with treated mice showing similar body proportions to healthy wild type (w/t) counterparts, which is a marked improved on the naso-anal length increase shown by CNP [25]. Again by tackling the problem at a root level there is much excitement over TA-46, though off-target effects remain to be seen.

It is hoped that TA-46 will act not just to increase vertical growth but bone thickness too. This could potentially mean increased vertebral foramen width thus decreasing significant morbidity caused by spinal stenosis. Pfizer recently acquired Therachon, thus TA-46 will be known as Recifercept [26].

### Pharmacological agents for Achondroplasia: Meclozine

Professor Matsushita of Nagoya University demonstrated through drug repurposing, symptom rescue in ACH transgenic mice treated for 3 weeks with Meclozine (a common anti-emetic) back in 2015 [27]. Meclozine showed improvement in total length and in body proportions. The authors predicted that in humans with ACH this treatment could equate to 6-7 cm height gain. Though Meclozine is a commonly prescribed drug, safety and pharmocokinetic data is limited to adults hence the need for clinical studies in children. Such a trial, in children aged 5–11 years (single arm, open, non-blinded, non-randomised) is in the recruitment phase (trial ID; UMIN000033052) [28]. The mechanism for how Meclozine drives growth in ACH is unclear though it is thought to be due to inactivation of FGFR3 signalling through inhibition of ERK (extra-cellular signal regulated kinase) to MAPK.

### Pharmacological agents for Achondroplasia: Infigratinib

QED Tx pharmaceuticals have developed Infigratinib, a pan-FGFR (1–3) TKI (tyrosine kinase inhibitor), more selective of FGF3 than other FGFRs, which has already shown meaningful clinical activity in cholangiocarcinoma patients. Infigratinib blocks tyrosine kinase activity of mainly the MAPK receptor (but also the STAT and SOX9 pathways) intracellularly and hence downstream signalling. It therefore counteracts FGFR3 hyperactivity. Phase 1 trials in ACH mouse models have shown improvements in both axial and appendicular skeletons, as well as correction in foramen magnum anomalies and vertebral disc defects, acting through not only the MAPK pathway but STAT and SOX9 (SRY-Box 9) too. Bone growth was reported as 2–3 times greater than as compared to BMN-111 and no other gross side effects were observed, probably since Infagratinib is selective of FGFR3 TKIs as compared to FGFR1/2 [29] (Table 2).

**Table 2:** Summary of treatment advances for Achondroplasia

Drug (Developer)	Type	Mechanism	Route	Phase
Vosorotide (Biomarin)	Small molecule peptide (CNP analogue)	Acts through NPR2 receptor to block MAPK pathway	Subcutaneous daily injection	3
TransCon CNP (Ascendis Pharma)	Small molecule peptide (CNP analogue) with TransCon carrier	Acts through NRPB1 receptor to block MAPK pathway	Subcutaneous weekly injection	2 (Q3 2019)
TA-46 (Therachon AG) soon to be known as Recifercept (Pfizer)	FGFR3 receptor decoy	Decreases FGFR3 activation	Oral	1 (2018)
Meclozine	H1 antagonist	Unclear (?MEK-ERK inhibition)	Oral	Preclinical studies completed
Infigratinib (QED therapeutics)	Small molecule TKI inhibitor	FGFR3 selective TKI	Oral	Preclinical studies completed.

It is an exciting time for Achondroplasia treatment, from no drugs to several candidates and the potential for combination therapy in the future.

### High bone mass conditions (Sclerosing bone disorders)

Healthy skeletal remodelling involves the intricate interplay between osteoblast-led bone formation and osteoclast-led bone resorption. Osteoblast and osteoclast activity is influenced and regulated by osteocytes [30].

### Osteopetrosis

Osteopetrosis (“marble bone” or “stone bone” disease) is the term used to describe a collection of rare, heritable skeletal disorders characterised by increased bone density on radiographs. They are most commonly inherited in an AD (autosomal dominant) fashion but can be autosomal recessive (AR) or X-linked (XL) [31]. The AD, AR and XL forms of osteopetrosis are predominantly caused by mutations in the *CLCN7, TCIRG1* and *IKBKG* genes respectively. There are several other genes including those that enable osteoclasts to acidify skeletal matrix.

Osteopetrotic conditions are broadly classified into 3 distinct forms based on age of onset and clinical features; infantile, intermediate and adult onset. Within each type, several different variants and molecular causes have been identified. Other rare forms exist that fall outside these categories (e.g. lethal, transient, acquired osteopetroses) and distinct forms associated with other disease such as renal tubular acidosis are also described.

**Fig. 2:**
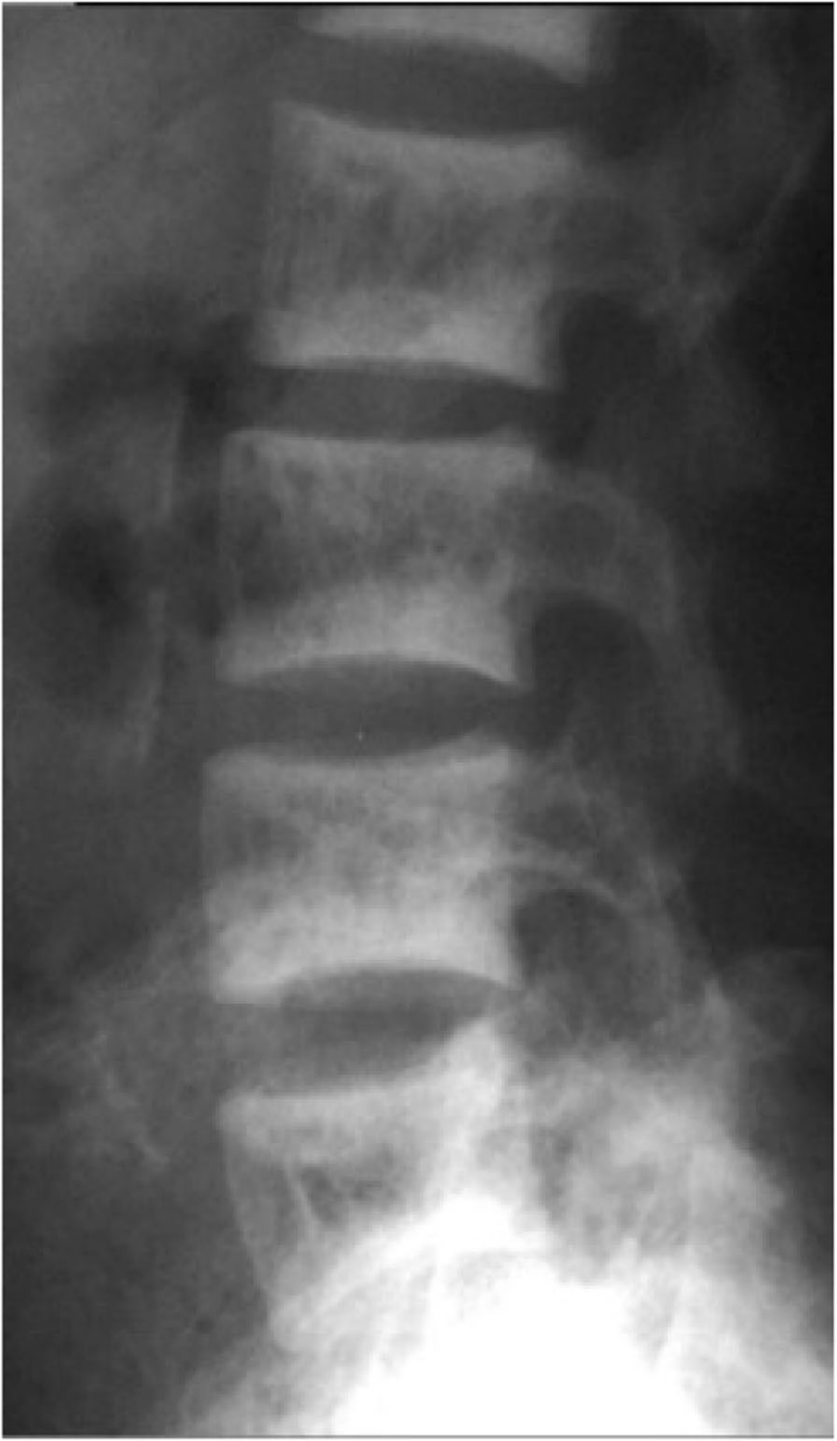
Rugger Jersey Spine in Osteopetrosis. Images taken from radiopedia.org under creative commons license 3.0 [32]

The infantile form tends to be recessive (ARO – autosomal recessive osteopetrosis) and the most severe form, frequently fatal in the first decade. The adult form is often dominantly inherited (ADO- autosomal dominant osteopetrosis), though is relatively benign with a good prognosis. The intermediate form is often recessive with a poor prognosis and present in childhood (Fig. 2).

Osteopetrosis is characterised by increased bone mass (dense) of poor quality in all compartments.

The underlying defect may lie in the osteoclast lineage itself (where osteoclasts can be low in number thus termed ‘osteoclast-poor’) or in the mesenchyme cells that form and maintain the microenvironment required for proper osteoclast function (where osteoclast number can be high, i.e. ‘osteoclast-rich’ osteopetrosis, where the osteoclasts are unable to secrete acid) [33].

Osteoclasts break down bone tissue during bone remodelling, a normal dynamic process whereby old bone is replaced with new bone to maintain bone health and mineralisation. In osteoclastic failure, old bone is not broken down as new bone is formed, thus bones become structurally abnormal, unusually dense and prone to fractures. Bone modelling (shaping) is particularly important during childhood (during the period of linear growth). Decreased tubulation of long bones by osteoclasts leads to widened metaphyses (club shaped bones) or an “erlenmeyer flask” deformity (a funnel like extremity with reduced medullary cavities).

ADO can radiographically result in the classic rugger jersey spine appearance (where vertebral endplates are densely sclerotic giving a ‘sandwich appearance’ (Fig. 2) or a bone within bone appearance [32].

In ARO the skull foramina fail to enlarge often leading to cranial nerve impingement. The sclerotic bone compromises marrow space leading to bone marrow failure; anaemia, and recurrent infections. Some patients have seizures due to hypocalcaemia due to diminished bone resorption. Skeletal features include osteoarthritis, fractures and osteomyelitis.

In XL osteopetrosis bony features can be associated with severe immunodeficiency and ectodermal dysplasia like features [34].

Though bone is more dense, it is still prone to fractures due to alteration of the bony architecture (decreased hydroxyapatite), and persistence of unresorbed primary spongiosa formed during enchondral bone formation (which appear as ‘bars’ of calcified cartilage within trabecular bone and are pathognomonic) [34].

### Treatments considered for osteopetrosis

Bone marrow transplant (BMT) is frequently an effective form of treatment for the recessive infantile form of osteopetrosis. BMT modifies the conditions natural course, as osteoclasts originate from the bone marrow (from monocytes). Bone marrow treatment, though curative in most cases is limited in availability with acceptable donors found in only 40% of cases, additionally BMT is associated with acute rejection, graft versus host disease and veno-occlusive events [35].

Long term interferon gamma-1b therapy (INFG, a cytotoxic and immunomodulatory agent) has also been shown to be effective and shows good haematological recovery though is not a cure hence is seen as a bridge to definitive BMT therapy. Key et al., described “treatment with INFG1b for 18 months stabilised / improved the conditions of all 11 patients treated for the full treatment period, and none of the 14 patients treated for at least 6 months died.” They showed additional benefit to white blood cell function, decreasing new infections. It is not exactly clear how INFG1b causes this improvement but it is hypothesised that INFG1b produces an osteoclastic superoxide which helps osteoclasts in degrading the bone matrix.

Other treatments for infantile osteopetrosis and less severe forms of osteopetrosis in which BMT is not indicated include;

- calcitriol which appears to stimulate dormant osteoclasts precursors into osteoclasts at low doses and at high doses stimulates RANKL production (despite initial promise, calcitriol use has fallen out of favour due to lack of clinical benefit in longer term use) [36].
- calcium and vitamin D (ergocalciferol / cholecalciferol) supplementation [35].
- erythropoietin, red cell transfusions and steroids to correct anaemia. The response to steroids is variable at best and has not demonstrated alteration in the natural course of the disease [38].

The evidence for these treatments is of either low or very low quality as assessed using the Delphi method by the Osteopetrosis Working Group in 2017 [37].

### Personalised treatment in ADO

ADO has two major forms; ADO Type 1 (ADO1) and ADO Type 2 (ADO2). ADO1 is typified by sclerosis (thickening) of the dome of the skull and is caused by gain of function *LRP5* gene changes. ADO2 is commoner than ADO1 and demonstrates the aforementioned ‘rugger jersey’ spine appearance and endobones (bone in bone appearance) in the pelvis. The majority (70%) of ADO2 cases are due to *CLCN7* gene dominant negative mutation. The *CLCN7* gene (chloride channel 7) codes for the hydrogen / chloride exchange transporter which is necessary for osteoclast acidification.

HSCT (haematopoietic stem cell transplant), a high risk procedure, is usually reserved for severe forms of ARO rather than milder ADO forms. Nevertheless patients still suffer lifelong severe symptoms thus a more personalised therapy is required.

It was proposed that short interfering RNAs (siRNAs) technology could work to knock out the affected allele. SiRNAs are produced within cells but can be introduced artificially. SiRNAs work by binding various proteins in the cell to target specific mRNAs (messenger RNA), which they degrade. In this case the siRNA targets the mRNA produced by the affected ADO2 allele and does not interfere with the normal wild type mRNA. Since ADO2 causes dominant negative mutations, neutralising the mutated mRNA induces a pseudo haplo-insufficient state, rescuing the phenotype [38]. Early studies on human mutant osteoclast cell lines have shown promising results by Capulli et al. [39]. Osteoblasts also express the *CLCN7* gene but at much lower levels than in osteoclasts and hence bone formation is not affected when the *CLCN7* gene is altered. Capulli’s group did not observe off-target effects of *CLCN7*^*G213R*^, *CLCN3* and *CLCN5* transcripts, suggesting high treatment specificity.

Further translational with respect to siRNAs in the GSD field has been slow. The author proposes that this is probably due to the challenge of delivering the siRNA to osteoclasts in the body (a systemic treatment), which would most likely require an osteoclast seeking factor and packaging of the siRNA with an extracellular vesicle (e.g. a liposome). SiRNA have been hugely successful where disease is localised such as in retinal ocular disease. Another challenge is many siRNA therapies, though injectable, require frequent dosing. One way around this could be to direct therapy to the phases of greatest growth (childhood) when bone modelling and remodelling are very active and osteoclast bone resorption is crucial for bone shaping, growth and development. Another way to overcome frequent dosing is to develop lentiviral vectors which lead to stable expression like with nusinersen (Spinraza), a recent genetic viral therapy for spinal muscular atrophy which we examine further in the discussion.

Interestingly CLC7 pump protein inhibitors can help increase bone mass and may show promise in bone loss conditions.

### Insights from ARO

A rare recessive form of osteopetrosis that provides important therapeutic insights is RANKL (Receptor activator of nuclear factor-kappa B (NF-κB) ligand, dependant osteopetrosis (RANKL-dependant ARO) caused by genetic defects to the function of the RANKL cytokine.

RANKL is a type II transmembrane protein otherwise known as osteoclast differentiation factor (ODF). Along with M-CSF (macrophage colony-stimulating factor), it is the master cytokine driving osteoclast differentiation and osteoclastogenesis. Defects in the *RANKL* gene prevent osteoclast formation and decreased bone resorption, leading to highly dense bone. Over activation of this pathway leads to osteoporosis, Paget’s disease and cancer related osteolysis [39, 40].

RANKL-ARO accounts for around 3% of all forms of ARO [41]. Sadly, these patients, commonly experience neurological deterioration with age (due to bony compression causing hearing / visual impairment), severe growth retardation (height well below the 0.4th centile), as well as worsening dentition. They also experience multiple poorly healing fractures, anaemia, thrombocytopaenia and recurrent infections. Several patients suffer upper airway obstruction leading to the need for positive airway support or even a tracheostomy. The severity of the condition demonstrates the need for targeted RANKL treatments. RANKL is highly expressed in mesenchyme cells not haemopoietic osteoclast lineages so is not amenable to HSCT.

Iacona and colleagues (2012) administered RANKL protein into mice with homozygous defects in RANKL, showing bone rescue (increased bone resorption) over a 1-month treatment period (with additional improvement in haemopoietic bone marrow function and improvement in splenic and thymic architecture) [41]. Unfortunately the promise of these early mice studies has not translated into further human based therapy as yet, as the higher doses in animal models led to cytokine overproduction and lethality secondary to respiratory failure [41].

Although personalised therapies have shown initial laboratory based promise in osteopetrosis more research is needed before translation to humans.

### Low bone mass conditions

#### Osteoporosis-Pseudoglioma syndrome

Osteoporosis-Pseudoglioma Syndrome (OOPG) is a very rare AR condition, with around 50 cases reported, caused by loss of function mutations in the *LRP5* gene [38]. OPPG involves severe osteoporosis and impaired vision. Visual loss is usually present at birth or early infancy and most patients are blind by young adulthood. The eye changes resemble a retinal glioma. The osteoporotic changes are usually noted in childhood leading to brittle bones and fractures, short stature, kyphoscoliosis, craniotabes and limb deformities [42]. Rare associated symptoms include mild intellectual disability, hypotonia, abnormally flexed joints or seizures.

Standard treatment in OPPG revolves around bisphosphonates which help bone strength somewhat but have not prevented bone fractures in the OPPG group [38].

Osteoclast mediated bone resorption occurs through resorption of hydroxapetite (a crystalline complex of calcium and phosphate) - the major component of calcified bone, which gives its rigidity and hardness [43]. Bisphosphonates are readily taken up the skeleton and contain pyrophosphate which selectively binds to calcium crystals (e.g. hydroxyapatite) preventing there growth and osteoclasts mediated dissolution, hence bisphosphonates increase bone mineral density (BMD). Though BMD is increased in OPPG this has not been shown to decrease fractures in OPPG.

Lithium is a well-known drug, used safely and effectively for over 50 years in psychiatric disease (specifically bipolar illness) and is known to strengthen bone and reduce fractures in the psychiatric cohort. An OPPG mouse model trialling Lithium showed dramatic improvement in bone strength and fracture prevention [44]. Streeten et al. devised a pilot study to explore the effects of 6 months lithium carbonate treatment on 10 OPPG patients. Only 2 patients continued the trial (of 5 in the intervention arm) and despite improvement in bone measurements these were statistically insignificant [45].

Though the trial failed, mouse studies showed much promise and postulated that Lithium worked via activation of the canonical WNT (wingless-related integration site) signalling pathway, downstream of the LRP5 (low density lipoprotein 5) receptor.

LRP5 and LRP6, along with Frizzled, are co-receptors of WNT, that combine to form the LRP5/LRP6-WNT-Frizzled complex. The activated complex leads to nuclear accumulation of β-catenin, increasing target gene expression, leading to increased bone growth. Lithium is known to inhibit glycogen synthase kinase 3β (GSK), an enzyme that phosphorylates cytoplasmic β-catenin, targeting it for ubiquitination and degradation. Hence Lithium leads to bone growth [38].

Another mechanism that has been explored is SOST (sclerostin) inhibition. SOST antagonises WNT signalling by binding to the extracellular domain of LRP5 and LRP6.

Anti-sclerostin therapy effectively removes the brakes on signalling through LRP5 and LRP6. Kedlaya et al. showed that inhibiting sclerostin with sclerostin antibodies in OPPG mice (Lrp5(−/−) led to osteoanabolic responses as shown by increased bone mineral density, content and formation rates. It is suggested that humans may benefit from similar treatment [46].

#### Osteogenesis Imperfecta

We briefly discuss the evolving OI treatment landscape, which is more expertly considered in the cited literature. OI is a rare heterogeneous genetic dysplasia that can be inherited in different ways (though AD is the commonest form, followed by AR, then XL). The hallmark of OI is weak bones that break despite little or no trauma, with dentinogenesis imperfecta, blue sclera and adult-onset hearing loss. Weak bones lead to bowing of the arms or legs, bone pain, fatigue, curvature of the spine, lax joints, muscular weakness, long term immobility and short stature. Clinical severity varies with the most severely affected individuals dying prenatally to those who have normal lifespans’, stature, dentition and only mild predisposition to fractures. Between 85 and 90% of OI cases are caused by mutations in the COL1A1 and COL1A2 genes which code for type 1 collagen (the most abundant collage in the bone, skin and other connective tissues) [47]. Other genes are implicated in rare types of OI, often encoding proteins involved in post-translational modification of collagen and genetic defects in osteoblast development for example; *CRTAP* (type VII OI) and *P3H1* (type VIII OI). OI type XIII (AR, *BMP1* gene) is unusual as it leads to high bone mass, though impaired bone microstructure and compromised bone strength [48].

There are currently 19 different types of OI as per OMIM classification, whereas the 2015 Nosology of GSDs prefers the Sillence classifications’ four main subtypes determined by severity, phenotypic and radiographic data (rather than molecular data), as the Sillence classification is still heavily used for prognostication [49, 50]. OI types I, III and IV account for 72–77% of the total OI population [51].

#### Treatments

Current standard of care in several types of OI is supportive, focusing on treating fractures and maximizing mobility including bisphosphonates, physiotherapy, mobility aids and surgery. Alongside bisphosphonates, several others drugs are in trials; Denosumab (a RANKL inhibitor), Fresolimumab (anti TGF-β (transforming growth factor beta) antibody therapy), Teriparatide (a form of PTH, parathyroid hormone) and anti-sclerostin therapy.

Bisphosphonates do not build new bone or improve the quality of existing bone in OI. Rather they increase bone mineral density (BMD) and preserve existing bone / prevent bone mass loss, as they bind hydroxyapatite which is usually taken up by osteoclasts (where they exert their anti-resorptive effects). Despite improvement in BMD and QOL, the data whether bisphosphonates (either oral or IV) prevent fractures in adults with OI is conflicting and inconclusive, though in children prolonged intravenous Pamidronate treatment has been shown to initially decrease fractures [52]. A recent Cochrane review found there was no conclusive evidence of improved clinical status in those with OI treated with bisphosphonates [55]. There are also concerns about long term use and complication such as osteonecrosis of the jaw and atypical femoral fractures.

Teriparatide (TPTD, a human PTH analogue) on the other hand builds bone (by activating osteoblasts) and is used in some cases of OI (with particular benefit in quantitative collagen deficiencies, thus Sillence type 1 OI [53]. The TOPAZ trial led by Professor Ralston from Edinburgh, UK is currently recruiting patients to see if TPTD and Zolendronate combined reduce the risk of fractures in adults with OI [54]. There is no particular role for GH in OI, as the short stature is not due to GH deficiency, though GH is helpful in type I and IV OI [55].

Osteoporosis (which by definition is a bone mineral density score as compared to a young healthy control of 30 year age of < or = to 2.5 SD from the mean) complicates many cases of OI. Such patients, after evaluation using the FRAX® clinical risk calculator for fracture assessment may require bisphosphonate treatment, though some are unable to tolerate it due to side effects of reflux oesophagitis.

#### OI and RANKL inhibitors (Denosumab)

For children affected by OI with osteoporosis, Denosumab (an anti-resorption RANKL agent) has been shown to raise bone mineral density (BMD) without adverse effects and decreased fracture rates in a Japanese study, though there were only 8 participants and only 3 of these under 16 years [56]. This study was limited by lack of a control group, short observation period for some patients (though 5 patients had Denosumab for > 30 months) and its retrospective design.

Denosumab is marketed as Prolia® or XGEVA® in the USA and is licensed for post-menopausal women who are at high risk of fracture due to osteoporosis. Denosumab works by inhibiting RANKL. RANKL, is a precursor that is involved in maturation of the osteoclast. By blocking RANKL, Denosumab blocks osteoclast maturation. In contrast, bisphosphonates work by binding bone mineral (in locations where minerals are absorbed by mature osteoclasts), induce osteoclast apoptosis and suppress resorption.

#### OI and anti-Sclerostin therapy (BPS804, Setrusumab)

We discussed the role of sclerostin and the mechanism for anti-sclerostin inhibitors when discussing the treatment of OPPG, a low bone mass condition.

Anti-sclerostin therapies are in development for OI too. Setrusumab (BPS804) developed by UK pharmaceutical Mereobiopharma, is an anti-sclerostin antibody recently evaluated as part of a phase 2A trial for moderate OI [57]. The phase 1A study, undertaken in the USA, Canada and Europe, involved 14 adult patients with OI (9 in the intervention group and 5 in the control). The intervention group received multiple escalating doses of BPS804 and were assessed at 6 weeks for bone biomarkers and at 20 weeks for BMD. BMD increased by 4% (*P* = 0.038) in the intervention group and was generally well tolerated. Results have been promising showing statistically significant improvement in BMD and bone biomarkers. A phase 2B randomised, double blinded control trial is currently in progress (the ASTEROID study) in the UK, USA and Europe, hoping to recruit 140 patients.

#### OI and TGF-β inhibitors (Fresolimumab)

TGF-β is a latent protein that awaits activation via osteoclastic bone resorption. Once activated and bound to its receptor, it induces smad and non-smad signalling [58]. TGF-β downstream effects vary. Osteoblast activity is reduced via different mechanisms. Osteoclasts maturation is inhibited (via RANKL / Osteoprogenitor) but conversely osteoclastogenesis is promoted through direct binding of TGF-β to osteoclasts. Thus overall TGF-β stimulation leads to more bone resorption than bone formation.

Fresolimumab is a monoclonal antibody that silences TGF- β. Studies in mice have shown that TGF-β increases bone mass, strength and quality [59]. Thus Fresolimumab has been suggested as a treatment for OI. A clinical trial in the USA is currently recruiting to evaluate the safety of Fresolimumab in adults with moderate to severe OI [60].

#### Stem cell approaches and regenerative medicine

BOOSTB4 is a European trial using stem cells to reduce morbidity in OI. Trial partners include the universities of Leicester, Utrecht, Lund, Cologne, the Karolinska institute, GOSH and UCL. Back in 2005, 6 children with OI were treated with adult MSCs (mesenchymal stem cells) post-natally in a US trial with promising results (increased growth, reduced fracture rates) [61].

The BOOSTB4 group felt that the use of fetal MSCs (rather than adult MSCs) and earlier intervention (in utero as opposed to post natal) could lead to better results based on research from animal studies. OI is most commonly diagnosed during the fetal anomaly ultrasound scan which is performed mid pregnancy in EU countries, thus antenatal treatment is possible. Fetal MSCs are felt to have greater colony-forming capacity and superior proliferative potential than adult MSCs thus greater therapeutic potential.

Since the US trial, researchers from within the BOOSTB4 consortium treated 4 more children ad-hoc using fetal MSCs (some post-natal, some antenatal). The children showed reduced fracture rates and improved growth [62]. By 2018 the 10 treated children were aged 3 to 16 years. Regulatory approval for the trial in Sweden was awarded in October 2018, and is currently in application in the UK and Germany. The trial has yet to start but one can keep up to date via the website [63]. It aims to recruit 15 families with foetuses affected by OI and infuse them with MSCs during the 2nd-3rd trimester, as well as another 15 who will receive MSCs after birth. All participants are to be followed for 10 years [64].

Fetal stem cell transplants are not new, with similar treatments attempted since the 1980’s and for other conditions (thalassaemia, sickle cell, severe immune deficiency), though responses have been mixed. Should this trial be successful it opens a whole new world for treatment of many different disorders. Despite this, stem cell approaches in OI do not aim to cure, since the patient will still have their own defective bone cells mixed with infused unaffected bone cells. Additionally there is concern that the MSCs proliferate into unwanted cancer cells.

Thus the door is open for further technologies dealing with the root genetic change. Hence approaches such as constructs to inactivate COL1A1 have also been explored, especially with new technologies; prime editing, CRISPR-CAS, TALEN and zinc finger nucleases [65]. Silencing of the dominant allele in OI would result in a milder phenotype and attempts using siRNAs, shRNA (short hairpin RNA) and antisense oligodeoxyribonucleotides (ODNs) have been promising, though mainly invitro and ex vivo. Besio et al. gives a comprehensive overview of recent gene and cellular therapy approaches [66]. Raloxifene (an oestrogen receptor modulator) is another experimental treatment shown to decrease fracture rates in mice (2016), though no human trials have been reported [67].

#### Hypophosphatasia (HPP) and X-linked hypophosphatemia (XLH)

It is important to note that the ‘osteoblast / osteoblast / osteocyte’ triad is not the only mechanistic pathway to low bone mineral density. Here we discuss mechanisms of defective bone mineralisation leading to low bone density conditions.

HPP and XLH are both rare genetic skeletal conditions. Though similarly sounding they are quite different. Biochemically HPP leads to markedly low levels of alkaline phosphatase (ALP) (raised or high normal plasma calcium and phosphate, low PTH) whereas XLH leads to low levels of phosphate. The underlying problem in HPP is inactive ALP which in turn leads to poor bone mineralisation. In XLH the kidneys are unable to resorb phosphate as normal, leading to low serum phosphate levels, thus lack of a key building block for bone formation.

#### HPP

Hypophosphatasia (HPP) is multi-systemic metabolic dysplasia inherited in autosomal dominant or recessive fashion, caused by loss of function mutations in the *ALPL* gene which encodes the tissue non-specific alkaline phosphatase enzyme (TNSALP) leading to defective bone mineralisation [68]. TNSALP is highly expressed in several different tissues; bones (in osteoblasts / chondrocytes), liver, kidneys and teeth, hence the ‘non-specific’ title.

**Fig. 3:**
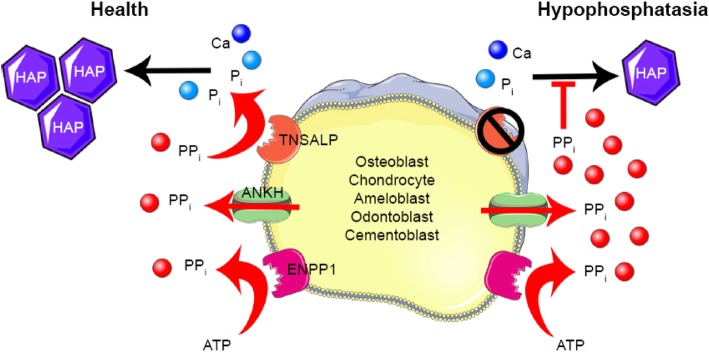
HPP mechanism of disease. Image used under the creative commons attribution license 3.0 from Bowden et al. [69]

Reduced phosphatase (TNSALP) activity results in elevated serum inorganic pyrophosphate (PPi), the biochemical villain in HPP. PPi is a substrate of TNSALP and an inhibitor of hard tissue mineralisation, sometimes described as the body’s ‘natural water softener’. High levels of PPi (substrate accumulation) block hydroxyapatite crystal growth, resulting in impaired bone or tooth mineralisation and thus rachitic symptoms [69] (Fig. 3).

There are 6 clinical forms of HPP (perinatal severe, perinatal benign, infantile, childhood/juvenile, adult and odontohypophosphotasia) [70]. Residual activity levels of TNSALP and age of onset correspond with levels of disease severity. Though one needs to be careful as the benign form can look very serious inutero but runs a mild course.

The dominant and adult forms of HPP tend to run a milder course, whereas the perinatal natal and infantile forms tend to be more severe due to respiratory complications resulting from narrow chest and pulmonary hypoplasia, and often the bones are extremely difficult to visualise radiographically [71]. Children with HPP tend to have soft abnormally shaped skull bones, short stature, bowed legs / knock knees, enlarged wrists and ankle joints, and hypercalcaemia which itself can lead to recurrent vomiting and kidney problems. Teeth can be lost prematurely (both primary and adult) and recurrent fractures heal poorly and can cause chronic pain. Craniosynostosis and vitamin B6 responsive seizures are frequent features in HPP. In adults findings are more subtle with crystal arthropathy, osteoporosis and recurrent fractures.

Previous treatments for HPP were symptomatic (low calcium diet) or involved IV infusion of ALP rich plasma from patients with Paget’s or purified placental ALP, teriparatide ‘off-label’ and bone marrow transplantation. These strategies have showed mild disease improvement but have been ineffective overall. Bisphosphonates are contraindicated as they are chemically analogous to PPI (which accumulates in HPP). The emergence of Asfotase Alfa (AA) has been revolutionary in the field [69].

AA (developed by Alexion pharmaceuticals) is a recombinant glycoprotein that contains the catalytic domain (active site) of TNSALP (i.e. hence is an ERT, enzyme replacement therapy) and therefore enables proper degradation of PPi. AA is administered subcutaneously in patients with perinatal, infantile, and juvenile-onset HPP. It gained FDA and European approval in 2015. Since its introduction several clinical trials and case reports have illustrated dramatic improvement in infantile HPP with reversal of skeletal mineralisation defects and improved survival [72]. AA has also shown improvement in quality of life / mobility in children and adults as well as improved growth in children [69].

Following the impressive results in severe forms of HPP, some suggest that AA should be considered in adult HPP under certain scenarios that are attributable to HPP; “musculoskeletal pain requiring prescription pain medications, disabling polyarthropathy, disabling functional impairment, low-trauma fractures, poor fracture healing, repeated orthopaedic surgeries for HPP and low BMD *T*-score in certain scenarios” [73]. The cost benefit ratio would need rigorous evaluation before such suggestions were conducted, and currently the evidence base for such strategies is very limited.

So far results from AA clinical trials have shown very good safety profiles with the main side effect limited to injection site reactions with marked scarring. Currently, all patients develop anti AA antibodies but no tachyphylaxis (diminishing response to successive drug doses). AA may potentially be an agent for other conditions that lead to abnormal accumulation of PPi such as neurofibromatosis type 1.

Despite these results there are several drawbacks with AA. Injections are frequent (3–6 times per week), cost, injection site reactions, possible long term tachyphylaxis and lifetime requirement of the drug. Thus alternatives to AA are still in development.

#### Alternatives to AA

Gene therapies using lentivirus (LV) and adeno-associated virus delivery of TNASLP have shown impressive results with single injections in animal studies, though these have not been translated in clinical human studies [74].

Interestingly BPS804 (a full human anti-sclerostin antibody) was trialled (phase IIA) in 8 adult patients affected by HPP in 2017 and showed promising results. SOST is an extracellular inhibitor of the Wnt pathway, thus downregulates osteoblastic bone formation, SOST inhibitors are therefore osteoanabolic. The authors considered that besides substrate accumulation (PPi) and deficient mineralisation, HPP is also marked by low bone turnover (through purinergic signalling) thus there is reduced bone formation [75]. By using BPS804 the study team hypothesised that sclerostin antagonism would lead to improved bone formation, increased BMD, upregulation of TNSALP and bone specific ALP.

The 8 patients were given escalating doses of the drug on 3 occasions over a month, and the followed up over 4 months. Results showed increased serum ALP enzymatic levels (including bone specific ALP), decreased bone resorption markers (C telopeptide of type 1 collagen, CTX-1) and increased bone formation markers (procollagen type 1 N-terminal propeptide, PINP, osteocalcin and PTH) [76]. PPi was not assessed as there is no assay for evaluating PPi in human samples. Results were similar to those observed in patients with osteoporosis treated with anti-sclerostin therapy. The increase in ALP was well below the increase seen in healthy subjects. Indicating the molecular defect cannot be overcome by anti-sclerostin treatment but can be ameliorated.

#### XLH

XLH, also known as vitamin D-resistant rickets is the commonest heritable and non-nutritional form of rickets, with an estimated incidence of 1 in 20,000 live births (from a Japanese study) and prevalence of 1 in 60,000 (from a Norwegian study) [77, 78]. XLH is a X linked dominant bone dysplasia caused by *PHEX* gene (phosphate-regulating gene with homologies to endopeptidases on the X chromosome) mutations. *PHEX* encodes the PHEX protein which regulates the FGF23 (fibroblast growth factor 23) hormone which is secreted from osteocytes. Normally FGF23 inhibits the kidneys ability to reabsorb phosphate to the blood stream and degrades vitamin D (1, 25-dihydroxyvit D) hence patients have low serum phosphate, high urinary phosphate and low or inappropriately normal serum 1,25-dihydroxyvitamin D levels. Classically boys are more severely affected then girls.

Inactivating mutations in the PHEX gene lead to FGFR23 over activity via mechanisms that remain incompletely understood.

The abnormal phosphate handling leads to phosphate wasting (high urinary phosphate) and insufficient phosphate for bone remodelling, thus weak and soft bones leading to bowed legs, bone pain, short stature and delayed walking. XLH usually manifests in childhood and is also associated with hearing loss, craniosynostosis, and dental abnormalities. By adulthood as disease progresses, patients can experience worsening bone deformity, bone pain, pseudofractures, enthesopathies and arthritis.

According to NICE, there are approximately 250 patients with XLH in the UK, though we assume this is an underestimate based on the earlier discussed Norwegian prevalence data, which if mapped to the UK population would suggest around 1000 cases [79]. Conventional therapy includes daily oral phosphate and activated vitamin D replacement and if started early does improve height, reduces bowing and the need for corrective surgery, though conventional therapy does not completely reverse the skeletal mineralisation defects, nor enthesopathies, and dental manifestations. Conventional therapy also requires close therapeutic monitoring to avoid hypercalcaemia, hypercalciuria, nephrocalcinosis, nephrolithiasis, and secondary or tertiary hyperparathyroidism. Phosphate supplementation (× 3–5 /day) is associated with adverse GI side effects; bloating, offensive stools, abdominal pain and urgency.

#### New personalised treatments in XLH targeting FGF23

In April 2018, the FDA approved Burosumab (Crysvita), an anti-FGF23 fully human monoclonal antibody, the first treatment to target the underlying pathophysiology of XLH. It was approved in Europe in February 2018. Crysvita increases the levels of the sodium phosphate co-transporter in the proximal kidney tubules and increases 1 alpha hydroxylase (the enzyme that coverts 25 hydroxylase to the active form of vitamin D).

NICE, in their draft guidance (mid 2018) rejected the drug on the grounds of cost, and uncertainty in the data around the benefits to young people aged 13–17 [80]. Subsequently, in September 2018, they approved the drug following a commercial arrangement with the company (Ultragenyx / Kyowa), making Crysvita provide ‘value of money’. Patients with PHEX mutations or a FH (family history) of XLH are eligible for Burosumab. Whilst NHS England try to implement the NICE guidance in full, Crysvita is being provided for eligible patients at no cost via the UK’s Early Access to Medicines programme.

Crysvita is taken as a two weekly injection from age 1 year until the skeleton stops growing. Peak bone mass is usually obtained in the third decade of life.

#### Metaphyseal dysplasia

The cost of developing a new drug in 2016 was estimated at $2.87 billion [81]. Experts suggest this is halved (or quartered) for rare diseases, as understanding of the molecular basis and related pathophysiology is often well described in rare diseases. Drug companies suggest profitability for orphan disease drugs is low citing small patient numbers. This argument is countered by that fact that the patient population is usually readily available, often spread across only 1–2 centres, thus decreasing the delivery pipeline cost. Also, drugs tend to be needed life-long, there is often a lack of competitors and orphan disease drug discovery can potentially lead to easy gains for other conditions.

Despite these arguments, investment in new orphan disease drugs is relatively low. Thus drug repurposing, has been a useful strategy. Carbamazepine (CBZ) is a well-known drug, used in epilepsy treatment for over 50 years, is being repurposed for patients with Metaphyseal Chondrodysplasia Schmid type (MCDS) and Schwartz-Jampel syndrome (SJS).

SJS is a very rare AR condition - AD cases have been reported - caused by *HSPG2* gene (Perlecan / Heparin Sulphate proteoglycan-2) mutations. Perlecan proteins are important in musculoskeletal development and are found in the pericellular matrix of chondrocytes. Key features of the condition include typical facial features (blepharophimosis, sad fixed facies, ptosis, small mouth with pursed lips, small high pitched voice) muscle hypertrophy and continuous myotonia. Skeletal abnormalities are often described but can be absent (short stature, scoliosis, joint contractures, bowing long bones, pectus carinatum, congenital hip dysplasia and metaphyseal dysplasia). Skeletal muscles are stiff and resistant to passive movement even at rest or sleep. Topaloglu et al. successfully showed improvement in myotonia for three SJS patients using CBZ in 1993. A further case report in 1997 demonstrated dramatic improvement in an infant’s skeletal symptoms post CBZ therapy, improvement felt to be due to relaxation of myotonia and therefore subsequent lessening of skeletal features [82].

MCDS is an AD condition caused by mutations in type X collagen, leading to short limb dwarfism. Mutations in type X collagen leads to misfolded collagen X, retained within the ER (endoplasmic reticulum) of hypertrophic chondrocytes causing increased ER stress. ER stress leads to the unfolded protein response and ultimately disruption of the normal growth plate and bone growth [83]. CBZ is able to reduce ER stress by stimulating intracellular degradation through autophagy or proteasomal degradation. This has been demonstrated in MCDS mouse models showing a reduction in severity of phenotypic features [84]. Michael Wright and colleagues in Newcastle are currently recruiting for a phase 1/11a investigator-led clinical trial, testing CBZ in ambulant MCDS children up to age 11 years (before growth plates fuse). UK recruitment opened April 2019 and 6 patients have been recruited so far (August 2019) with a further 6 spaces available for stage 1. The initial stage involves a 12 month observational study. This will be followed by a 12 month dosing stage at which point International partners will be eligible to recruit to. More information about the trial can be found at the trial website [85]. Diagnosis of MCDS is primarily made based on skeletal survey findings (metaphyseal abnormalities of upper and lower limbs, coxa vara, tibial and femoral bowing). Patients present with short bowed long bones, short stature, normal trunk length, no facial dysmorphism, waddling gaits and short broad hands.

#### Fibrodysplasia Ossificans Progressiva (FOP)

FOP is an extremely rare and severely disabling AD condition, in which damaged fibrous tissue (e.g. muscle) turns into bone, affecting 1–2 per million population. FOP is caused by activating mutations in the *ACVR1* gene (Activin A receptor, type 1), classically c.617G > A R206H mutations. This mutation leads to over activation of BMP-Smad1/5/8 signalling and promotes ectopic chondrogenesis and osteogenesis.

The Activin A receptor accepts several different ligands including Activin A (a non-canonical ligand) and other type 1 BMPs (bone morphogenic proteins) [86]. Activin A binding to mutant ACVR1 (R206H), activates SMAD 1/5/8 signalling, whereas binding to w/t ACVR1 leads to down-regulation of the SMAD 1/5/8 pathway [87, 80].

In FOP, muscle and connective tissue ossify during ‘flare-ups’, often due to tissue trauma but sometimes in its absence. The HO (heterotopic ossification or extra-skeletal bone growth) forms a second skeleton. This process begins in childhood, leading to joint dysfunction and whole-body movement constraint leading to feeding and breathing difficulties and premature death. Many patients are wheelchair bound by their third decade. Individuals affected by FOP tend to have congenital large halluces and short thumbs.

#### Treatments in FOP

The current standard of care includes glucocorticoids in the acute ‘flare-up’ setting but this does little to prevent subsequent HO. Surgical removal of HO results in extreme bone growth. Several studies are however under way assessing new treatments for FOP including Palovarotene, REGN2477 and rapamycin.

#### REGN 2477 (anti Activin A)

Activin A stimulates HO in FOP and is known to be released from innate immune cells, hence the link between inflammation and HO. Regeneron Pharmaceuticals showed that anti-Activin A (REGN 2477) showed significant efficacy in mouse studies at preventing HO and regression of HO lesions. REGN 2477 is currently in phase 2 trials (Clinicaltrials.gov registry NCT03188666). It is important to consider that Activin A is ubiquitously expressed and is important in neuronal differentiation and inflammation thus effects of anti-Activin A need careful monitoring.

#### Palovarotene (retinoid agonist, previously known as R667)

Retinoid signalling is important in chondrogenesis and proper skeletal formation [89].

Pre-clinical studies show that all-trans retinoic acid therapy leads to repression of chondrogenesis and cartilage formation (by blocking differentiation of chondroblasts). In FOP, HO proceeds through a cartilage intermediate via enchondral ossification. This explains why retinoids are an attractive target. But non-selective retinoid therapy is non-specific and has many side effects; including teratogenicity, fetal limb malformations. And muco-cutaneous side effects.

There are three retinoic acid receptors (RARs);α, β, and γ. RARγ is expressed in chondrocytes and mesenchymnal precursor cells [90]. Palovarotene is a highly selective RAR-γ agonist.

Animal studies in 2011 showed that RAR-γ agonists (including Palovarotene) blocked new bone formation in genetically modified ‘knock-in’ mice with FOP containing an active ACVR1 receptor [91]. By 2016 a multi-centre phase 2 clinical trial supported by Clementia Pharmaceutical was published, involving 40 subjects with average age, 22 years. Palovarotene decreased the percentage of patients with FOP who develop HO, improved flare-up resolution and decreased patient reported pain [92]. Palovarotene is currently in phase 3 trials.

#### Rapamycin

Rapamycin (Sirolimus), a relatively common drug, used for immunosuppression post-transplant surgery and to treat lymphangioleiomyomatosis in Tuberous Sclerosis.

Rapamycin targets mTORC1 (mechanistic target of rapamycin complex 1) and therefore multiple signalling pathways including HIF1α (hypoxia inducing factor 1 alpha). The mTOR pathway interacts with the RANKL/RANK system though it is not exactly clear how.

A drug screen of over 6800 substances on induced pluripotent stems cells (iPSC) grown to mimic FOP showed inhibited abnormal bone formation with rapamycin. The FOP iPSCs were transplanted into mice and bone inhibition was demonstrated.

Rapamycin has been used in two paediatric patients with FOP at pharmacological doses for 3 months and 18 years respectively (the latter to prevent Liver transplant rejection). In neither case did it show clinical response from a FOP point of view. Despite this a clinical trial evaluating its efficacy in FOP is underway at Kyoto University Hospital [93].

#### Emerging drug therapies in FOP

Imatinib, often used for chronic myeloid leukaemia, has been used off-label in several children with FOP with relentless flare-ups refractory to standard care [94]. Six of the seven showed dramatic improvements with limited side effects, though this was not a RCT. It is thought that Imatinibs’ effects in FOP are mediated through interaction of multiple pathways including HIF1α and MAP kinase signalling.

Another key strategy is to target the tyrosine kinase activity caused by the ACVR1 mutation through Saracatinib. Several drug companies are developing agents (BCX9250, BCX9499, and BLU-782) on the back of promising preliminary invitro and in vivo studies.

Another promising drug is LDN-212854 a BMP type 1 receptor inhibitor that has shown specificity for ACVR1.

In the long term drugs that target the underlying genetic error, gene overexpression – such as RNA interference, will prove a powerful method to combat the disease but as yet the challenges remains with delivery of such therapies to reach the target without unwanted off-target effects [95].

It is an exciting time in terms of drug development for FOP. Combinational therapy at multiple target levels would seem to be the future.

#### Genetic bone tumours

An often striking and relatively common presenting clinical feature in children is bony lumps / growths. If bony lumps are multiple or familial, then two important AD inherited diagnoses need consideration; HME (Hereditary Multiple Exostosis also known as Hereditary Osteochondromatosis) and Metachondromatosis (MC). HME has an estimated incidence of 1/50,000 live births affecting males and females equally, though males more severly. Fewer than 100 case of MC have been reported [96]. It can be difficult to distinguish the two but is more important than ever to do so, as trials of potentially disease modifying drugs in HME are under way. Additionally, HME lesions have greater malignant potential than in MC, and MC lesions often regress so surgery can be unnecessary, unlike in HME where patients may need multiple surgeries [97].

HME involves multiple exostoses (osteochondromas). Exostoses are cartilaginous capped bony growths that arise next to and grow away from the growth plate (they ‘never’ originate from the growth plate). MC involves both osteochondromas and enchondromas (intraosessous hyaline cartilage based lesions arising from the metaphysis or diaphysis). Enchondromas are often orientated towards the epiphyses rather than away [98]. Thus a bony growth arising mid-shaft may indicate an enchondroma, but presentations of lesions are not usually so obvious. Radiographs are key to identifying enchondromas but diagnosis is still difficult if bone lesions arise in the metaphyses. In such cases, MRI scans distinguish truly intraosseous enchondroma lesions, but one has to weigh up the anaesthetic risk for paediatric MRIs against the benefit of early diagnosis.

Another key ‘history’ distinguishing feature is ‘natural course’. MC lesions tend to regress by adulthood, whereas HME lesions often only plateau (around puberty) rather than regress, though new HME lesions do not to arise in adulthood. Lesions in HME / MC tend not to be painful themselves but can cause pain through pressure on local structures, in addition to nerve or vascular compression, reduced joint movement, bone deformity and cosmetic disfigurement requiring surgery [90]. Treatment is not always required in HME if the bony lesions are small. HME is caused by mutations in *EXT1* (69%) and *EXT2* (21%) genes (about 10% are denovo), and MC by activating mutations in the *PTPN11* gene [90] (Table 3).

**Table 3:** Summary of key bone tumour conditions and emerging therapies

	HME	MC	Ollier	Maffucci
Incidence	Relatively common (1 in 50,000)	Rare (< 100 cases reported)	1/100,000 (commonest enchondromatosis subtype) [99]	Rare (160 reported cases)
Inheritance	AD	AD	Sporadic / Somatic	Sporadic / Somatic
Genes	EXT1 and EXT2	PTPN11	IDH1 and 2, PTHR1	IDH1 and 2
Lesions	Osteochondromas (OCs)	Osteochondromas and enchondromas	Enchondromas	Enchondromas + vascular malformations
Key History	Lesions plateau in growth at puberty but do not regress. Lesions do not arise in adulthood. OCs form at long bone ends (forearm, femur) and on flat bones (hips, shoulders, blade or ribs)	Lesions resolve with time (by adulthood often) Lesions usually in hands and feet Enchondromas tend to arise from the metaphyses (so can grow into the diaphysis or growth plate), whereas OCs arise next to the growth plate and grow away from it.	Starts in 1st decade of life Enchondromas as described in MC. Tend to be one sided or localise to one area, especially hands over feet. Bones deform more severely than in HME/MC.	Like Olliers but also get venous malformations (VMs, often presenting as soft blue subcutaneous nodules that empty with pressue) and less commonly haemangiomas or lymphangiomas or phleboliths. Starts in1st decade of life, usually between age 4-5 years.
Gender	Males more severely affected.	Tends to affects males	Tends to affects females	Equally affects males and females
Malignancy risk	4% [100] (2–5%)	< 1%	25% (5–50%) Skeletal, brain, visceral, ovarian	30% (25–40%) [101] Skeletal and vascular
Trials	Phase 2 retinoic acid like treatment	–	–	Rapamycin for spindle cell VM (case report) but failed for haemangiomas (case report) [102, 103]

#### Retinoic acid as a treatment for HME?

A new international phase 2 clinical trial for HME called the MO-Ped trial (Clinicaltrial.gov identifier: NCT03442985) sponsored by Clementia pharmaceuticals completed patient enrolment in August 2018. The trial drug (Palovarotene) is an orally based selective retinoic acid treatment. Participants must have a confirmed diagnosis of HME with mutations in EXT1 or EXT2 and be between ages 2–14 years (confirmation testing can occur within the trial). 240 participants will be given different doses of Palovarotene by daily oral ingestion over a 24 month period [104]. Patients with contiguous gene deletions (Langer Gideon syndrome for *EXT1* and Potocki Schaffer syndrome for *EXT2* are not eligible for the trial).

HME causes osteochondromas (cartilage capped bony growths) thus enchondral ossification is the key bone formation method. As discussed in therapies for FOP, retinoids cause repression of chondrogenesis and cartilage formation (by blocking differentiation of chondroblasts). EXT1 and EXT2 both code for glycosyltransferase enzymes that are involved in the synthesis of heparin sulphate, a critical component of cartilage. Heparin sulphate is a key part of the matrix and important in regulating other signalling. Absence of heparin sulphate leads to local increase in BMPs and smads which ultimately leads to abnormal budding of osteochondromas at bone growth plates (exostoses).

Palovarotene (a RAR-γ agonist) has been shown to block new bone formation in genetically modified mice. Osteochondroma count is reduced by upto 91 and 92% in ribs and long bones respectively of HME mice (mean %), when considering the 1.76 mg/kg/d dosing regimen. Improvement was also seen in long bone length and histology [105].

Considering the potential teratogenic side effects of such therapy, females of child bearing age undergoing treatment would need to take highly effective methods to prevent pregnancy. Other concerns include the risk to mental health with an association described between isoretinoin and depression / suicide risk / long term mental health problems (when used in the treatment of acne).

#### Non-hereditary bony lumps; Ollier disease and Maffucci syndrome

Non-hereditary conditions such as Ollier disease and Mafucci syndrome (both caused by somatic mosaicism) are important differentials in patients presenting with bony lumps, especially as HME or MC can present denovo, so family history can be non-discriminatory. The key distinguishing features for Ollier’s is that the multiple echondromas are extremely variable in size and onset, tend to occur in extremities and present unilaterally or localised to one area. Radiographically, lesions tend to cluster and calcify / stipple with time [106].

If the enchondromas are non-hereditary and associated with vascular malformations (often venous but also haemangiomas) or lympahngiomas or phleboliths then the diagnosis of Maffucci syndrome should be considered. Maffucci syndrome usually presents in childhood in the extremities (with a preponderance of the hands over the feet).

One should also consider other differentials: Spondylo-enchondroplasia with immune dysregulation (SPENCDI, APC5 gene, recessive inheritance, which features autoimmune disorders, neurological sequalae and intellectual deficit) and genochondromatosis (gene unknown, AD, whereby enchondromas present throughout the body especially over the clavicles and knees [107].

## Discussion

In conducting this review, using Pubmed as our principle search database, we note its’ bias towards North American, European and Japanese publications as well as bias towards publishing studies with positive findings, limited inclusion of conference proceedings and ‘grey’ literatures. We partially overcame some of this bias by identifying conference proceedings from the 2016 American Society for Bone and Mineral Research annual meeting and 2017 ISDS (International Skeletal Dysplasia Society) conference and 2019 SDG (Skeletal Dysplasia Group) UK Treatments update symposium.

Change is the only constant in the evolving therapeutic landscape of skeletal disorders. In the last 15 years the pace of change has been rapid as the scientific community, with the aid of genetic advances, better understand the biological mechanisms of these rare but important conditions. It is impossible to summarise all the new knowledge gained from the study of rare skeletal disorders in even a review.

Van Buchem disease / sclerosteosis and the discovery of the SOST gene are a great example of novel gene identification leading to novel targets for common conditions. They led to the development of anti-sclerostin therapies for osteoporosis and other low bone mass disorders. Equally the discovery of CTSK (cathepsin) as the cause of pycnodysostosis has led to the development of a new class of bone resorptive drugs; CTSK inhibitors, which have been trialled for osteoarthritis. Ironically though these rare conditions in particular have unlocked therapies for common conditions, affected patients are still without treatment. This highlights the difficulty of drug development in rare disease where financial returns on invested resources can be deemed relatively minimal. Additional challenges for rare diseases apply, such as small patient groups, difficulty in measuring clinical end points and trials in paediatric populations. Strategies to overcome these challenges include readily identifiable patient populations, often limited to 1–2 national centres, motivated patient groups, lack of competing drugs, active research interest both by clinicians and academia, adaptive licenses for rapid trials and translational work and potentially life-long treatments. Nevertheless, it is important to continue to discover new genes for the many as yet undiagnosed and unexplained skeletal syndromes / phenotypes.

### Gene therapies the way forward?

The first gene therapy was developed in 1990 for recessive severe combined immune deficiency. Since then therapies have been developed for B-thalassaemia major, adrenoleukodystrophy, haemophilia, CLL, ALL, Leber’s congenital amaourosis (LCA) and Huntington disease amongst others [108].

Yet we have seen limited progress in terms of gene therapies for inherited bone disorders. Perhaps cost is the biggest obstacle. The cost of Luxturna gene therapy for LCA in both eyes (that leads to dramatic improvement in vision, but is not a cure) was reported as $850,000 in 2018. Though this price may seem astonishingly high, it is a one-off cost and if compared to the market price of other recent gene therapies like Nusinersen for spinal muscular atrophy - which costs$750,000 in the first year, then $350,000 per year thereafter - Luxturna is relatively cheap [109, 110]. It seems the cost of development of such drugs and the current technologies limit the development of gene therapies in the rare bone disorders field. Though if compared to standard treatments such as bone marrow transplants they may not be as expensive as thought. In 2017 there were 899 clinical trials involving gene based therapies worldwide, so no doubt progress is near [111].

## Conclusion

Inherited bone dysplasias though individually rare and often unique in terms of the causative gene, share similar pathophysiological and molecular pathways in altering the bone function of the body. The study of skeletal disorders has greatly expanded our understanding of normal skeletal physiology and bone signalling pathways, suggesting new targets and strategies for improving skeletal health. It has also led to the development and or repurposing of therapies for common and rare conditions,

In compiling this review we have come across a broad range of different strategies for therapeutic interventions in rare GSDs;

- treating osteoclastic over activity
- targeted enzymes (enzyme replacement therapies) and proteins e,g. HPP TNSALP
- monoclonal antibodies; Denosumab (RANKL inhibitor for OI and FOP), Setrusumab (sclerostin antibody in HPP and osteoporosis)
- small molecule approaches (e.g. peptides, CNP in Achondroplasia),
- protein decoys (TA-46, sFGFR decoy in Achondroplasia)
- cell based approaches (mesenchymal stem cells in OI, bone marrow cell transplant in osteopetrosis)
- gene based therapy
  - gene silencing
  - siRNA (RNA interference for CLCN7 ADO type 2) (Table 4)

**Table 4:** Summary table of conditions discussed and related treatments

	Disease	Treatments (stage if still in trials)	Additional Notes
FGFR3 spectrum disorders	Achondroplasia	Statins (in mouse models) GH Vosoritide (CNP analogue, phase 3) TransCon CNP (phase 2 (Q3 2019)) TA-46 (soluble FGFR3 ligand trap, Therachon AG, phase 1) Infagratinib (preclinical studies complete) Meclozine (drug repurposing, phase 1)	-Studies of Statin related therapy show conflicting results.
	Hypochondroplasia	Somatropin (human growth hormone)	
High Bone Mass	Osteopetrosis	Interferon gamma 1b Human stem cell transplantation siRNA therapy (in vitro cell lines) RANKL replacement (mouse models)	siRNA therapy: For AD Osteopetrosis RNAKL replacement: For RANKL AR Osteopetrosis
Low Bone Mass	Osteoporosis Pseudoglioma syndrome	Lithium carbonate (mice studies promising) Anti-sclerostin therapy (mouse model)	Lithium carbonate: -limited data from pilot human studies
	Osteogenesis Imperfecta	Bisphosphonates, GH Teriparitide RANKL inhibitors (Denosumab, clinical trials) Anti-sclerostin therapy (Setrusumab, BPS804) TGF-β inhibitors (Fresolimumab, phase 1) Stem cell (mesenchymal, inutero transplant) Raloxifene Gene silencing –siRNA, shRNA, ODNs Vibraflex device (BCH), Physical rehab training	GH: -GH in OI in type IV Teriparitide: -TOPAZ trial (Teriparitide and Zolendronate, recruiting) Setrusumab: -phase 2B trial (ASTEROID study) Fresolimumab: -phase 1 trial in US (recruitment) Stem cell: -BOOSTB4 trial (gaining trial approval) Gene silencing: -At invitro and ex vivo stage. Vibraflex: -conflicting evidence for benefit
Low Bone Mineral Density	Hypophosphatasia	Asfotase Alfa (approved) Gene therapies (animal studies) Anti-sclerostin therapy (BPS804, human proof of concept trials) Bone marrow transplant, Teriparatide	Asfotase Alfa: For perinatal, infantile, juvenile HPP
	-X-Linked Hypophosphataemia	Anti FGFR23 mab; Burosumab (Crysvita)	Approved by NICE Sept 2018
Metaphyseal Dysplasia	-Metaphyseal Chondrodysplasia Schmid type	Carbamazepine (phase 1–2, Newcastle led)	Recruiting 2019.
	-Schwartz Jampel Synd.	Carbamazepine (case reports)	
FOP	Fibrodysplasia Ossificans Progressiva	REGN 2477 (Anti Activin A, phase 2) Palovarotene (selective retinoid agonist, phase 3) Rapamycin Other small molecule therapies in development.	
Genetic Bone Tumours	Hereditary Multiple Exostoses	Palovarotene (phase 2)	

Whilst awaiting breakthroughs with cell and gene based approaches, repurposing of drugs has a major role in improving patient morbidity. We highlighted the useful approach of drug screening on cell lines or mouse models which not only highlighted candidate drugs, but provided clues to pathological mechanisms involved in the dysplasia. Key drugs in current clinical trials include;

- Carbamazepine (for MCDS, SJS), due to its role in ER stress.
- Lithium carbonate (for OPPG) due its role in WNT signalling activation.

Should these trials prove successful they may open the role for these drugs to be used in other skeletal disorders due to the broad mechanisms of action. For example lithium carbonate roles in WNT signalling may suggest it valuable in other conditions where WNT signalling is important such as HPP.

By considering the skeletal dysplasia as a group it allows one to consider the common mechanisms of disease and the usage of novel / effective therapies across several disease groups. Palovarotene (a retinoic acid agent) showed utility in clinical trials for FOP, a condition that causes heterotopic ossification, thus the drug is being trialled in HME, a condition that is defined by heterotopic ossification. When studying / treating a rare disease it is therefore useful to look at the related conditions and the emerging treatments for those conditions as this can unlock possible therapeutic options for the disease of interest.

For diseases that have very small numbers of patients it is opportunistic to study the effects of a common therapy on the multiple different disease cohorts to better appreciate the similarities and differences in clinical responses. For example, Denosumab (RANKL inhibitor) and its use in OI and FOP.

As our understanding of the mechanisms involved within the bone mineral unit, conversion of osteoblasts to osteocytes, maturation of osteoclasts and related mechanisms evolve, new osteoblastic, osteocytic and osteoclastic therapeutic targets will no doubt emerge.

We eagerly await the results of many of the discussed trials as successful results may prove to be fundamental for not only the studied skeletal dysplasia but for many other conditions. In particular the BOOSTB4 trial of in-utero mesenchymal stem cell transplantation for OI could open the use of this approach in many other rare diseases. Especially for conditions like RANKL related ARO since it would not benefit from HSCT (RANKL genetic defects originate from mesencyhmal cells not haemopoietic stem cells).

Novel approaches like induced pluripotent stem cells, may prove to be pivotal in development of drugs in rare disease. Professor Shinya Yamanka (Kyoto University) - winner of the 2012 Nobel Prize in medicine for discovering iPSC cells - hopes the Rapamycin trial in FOP will “spur active research for drug development, and eventually lead to the discoveries of new treatment and various rare diseases”.

Given the rarity of inherited bone disorders, for progress to accelerate greater collaboration between the different stakeholders; academic research centres, universities, charities, pharmaceutical companies, biotech industries, clinics, and hospitals, is required in order to overcome the safety and regulatory issues in developing new therapies for rare patient cohorts.

## Data Availability

not applicable.

## Abbreviations

AA: Asfotase Alfa
ACH: Achondroplasia
*ACVR1* gene: Activin A receptor, type 1
AD: Autosomal dominant
ADO: AD osteopetrosis
ALP: Alkaline phosphatase
AR: Autosomal recessive
ARO: AR osteopetrosis
BMD: Bone mineral density
BMN111: Vosoritide
BMN250: Tralesinidase alfa
BMPs: Bone morphogenic proteins
BMT: Bone marrow transplant
CBZ: Carbamazepine
cGMP: Cyclic gaunosine-3′.5′ monophosphate
CNP: C-type natriuretic peptide
CTX-1: C telopeptide of type 1 collagen
ECM: Extracellular matrix
EMA: European Medical Agency
ER: Endoplasmic reticulum
ERK: Extra-cellular signal regulated kinase
ERT: Enzyme replacement therapy
FDA: US Food and Drug Administration agency
FGF23: Fibroblast growth factor 23
FGFR3: Fibroblast growth factor receptor 3 gene
FH: Family history
FOP: Fibrodysplasia Ossficans Progressiva
GSD: Genetic skeletal disorders
HCH: Hypochondroplasia
HIF1α: Hypoxia inducing factor 1 alpha
HME: Hereditary Multiple Exostoses
HO: Heterotopic ossification
HPP: Hypophosphotasia
HSCT: Haematopoietic stem cell transplant
HSPG2 gene: Perlecan / Heparin Sulphate proteoglycan-2
INFG: Interferon gamma-1b therapy
iPSCs: Induced pluripotent stems cells
LCA: Leber’s congenital amaourosis
LRP5: Low density lipoprotein 5
LV: Lentivirus
MAPK: Mitogen-activated protein kinase
MC: Metachondromatosis
MCDS: Metaphyseal Chondrodysplasia Schmid type
mRNA: Messenger RNA
MSCs: mesenchymal stem cells
mTORC1: Mechanistic target of rapamycin complex 1
NAGLU: N-acetylglucosaminidase
NRP B: Natriuretic-peptide receptor B
ODF: Osteoclast differentiation factor
ODNs: Antisense oligodeoxyribonucleotides
OI: Osteogenesis Imperfecta
OPPG: Osteoporosis-Pseudoglioma Syndrome
PHEX gene: Phosphate-regulating gene with homologies to endopeptidases on the X chromosome
PINP: Procollagen type 1 N-terminal propeptide
PPi: Inorganic pyrophosphate
PTH: Parathyroid hormone
RANKL: Receptor activator of nuclear factor-kappa B (NF-κB) ligand
RARs: Retinoic acid receptors
RNA: Ribonucleic acid
sFGFR3: Soluble FGFR3 protein
shRNA: Short hairpin RNA
siRNAs: Short interfering RNAs
SJS: Schwartz-Jampel syndrome
SOST: Sclerostin
SPENDCI: Spondylo-enchondroplasia with immune dysregulation
STAT: Signal transducer and activator of transcription
TD: Thanatotrophic dysplasia
TGF-β: Transforming growth factor beta
TKI: Tyrosine kinase inhibitor
TNSALP: Tissue non-specific alkaline phosphatase enzyme.
TPTD: Teriparatide
w/t: Wild type
WNT: Wingless-related integration site
XL: X-linked
XLH: X-linked hypophosphatemia
